# ZrO_2_-based heterojunction engineering for enhanced visible-light photocatalysis: a review

**DOI:** 10.1039/d6ra06664j

**Published:** 2026-09-07

**Authors:** Sivasundhari Sivakumar, E. James Jebaseelan Samuel

**Affiliations:** a Department of Physics, School of Advanced Sciences, Vellore Institute of Technology Vellore 632014 Tamil Nadu India ejames@vit.ac.in

## Abstract

Rapid industrialization has significantly increased water pollution worldwide, requiring sustainable and efficient technology for water treatment. Visible-light-assisted photocatalysis is one of the promising techniques that can be employed for environmental remediation by oxidizing various pollutants through the use of solar energy. Zirconium dioxide (ZrO_2_) is an environmentally friendly and chemically stable compound with high oxidative power, but it suffers from a wide bandgap that inhibits the utilization of visible light. The fundamental principles of photocatalysis, intrinsic properties and limitations of bare ZrO_2_, heterojunction formation approaches, synthesis methods, characterization analysis, and photocatalytic mechanism have all been discussed in this critical review of current progress in ZrO_2_-based heterojunction photocatalysts for water purification. Issues related to stability and recyclability of catalysts, charge carrier behaviour, photocatalytic activity, and the influence of test conditions have been critically analysed. This review highlights the importance of standardization of photocatalytic experiments, testing of stability, evaluation under realistic wastewater systems, and complete investigation of mineralization and energy efficiency. In order to rationalise the development of robust, efficient, and practical ZrO_2_-based heterojunction photocatalysts for sustainable water purification, current knowledge gaps and future perspectives have been highlighted.

## Abstract 2

ZrO_2_ based heterojunction engineering enhances visible-light absorption and charge separation, improving photocatalytic pollutant degradation for sustainable water purification.

## Graphical abstract

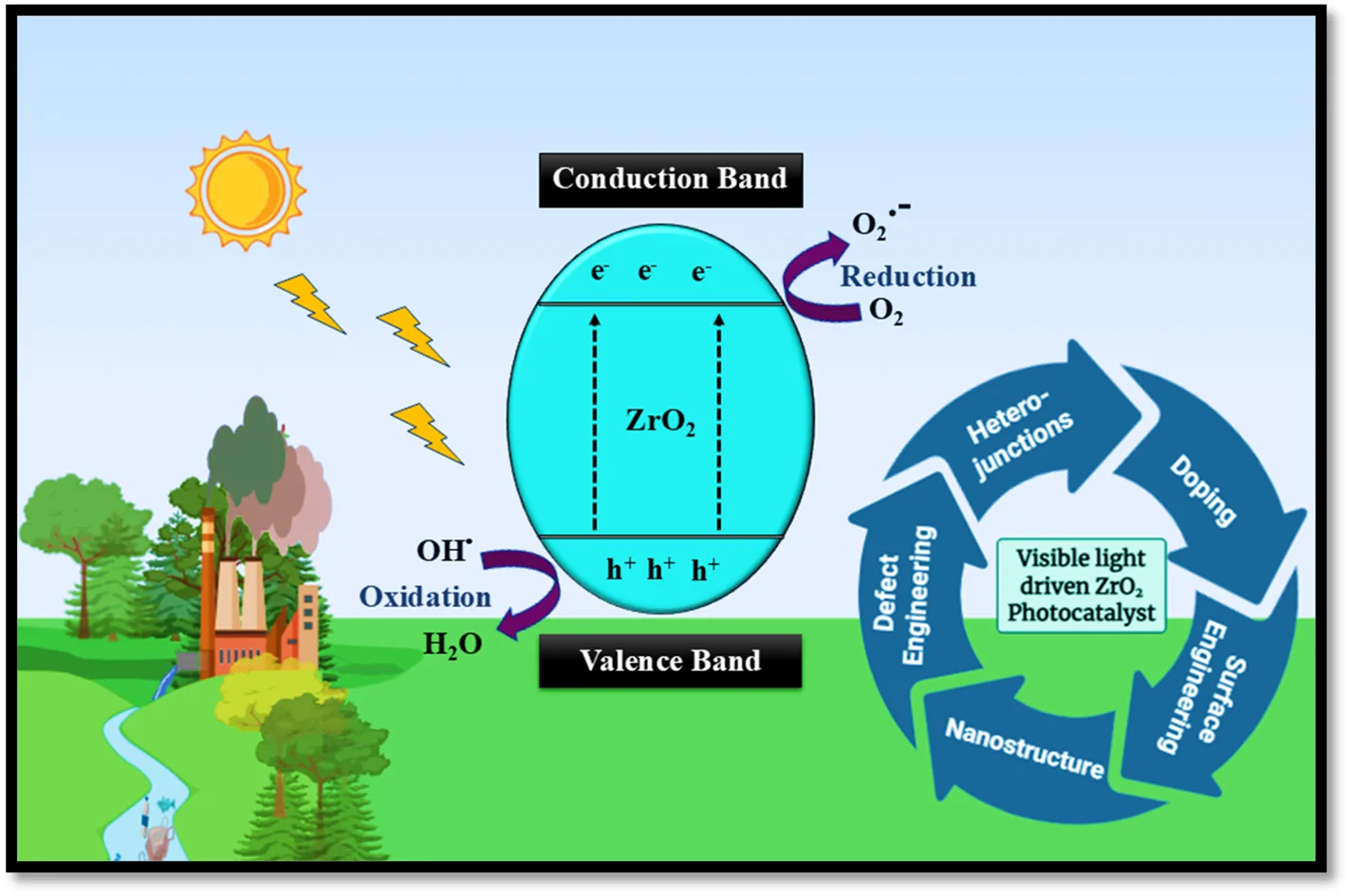

## 1. Introduction

Water plays a crucial role in sustaining life and protecting the environment; nonetheless, economic development faces a serious impediment due to the declining supply of clean water that has been further exacerbated by pollution caused by industries.^1,2^ Organic dyes commonly find their way into water supplies through the dyeing process and have found widespread applications within sectors like papermaking, textile manufacturing, and pharmaceuticals.^3^ Organic dyes, particularly azo dyes, are difficult to remove from water; they have the potential to contaminate both surface water and underground water sources. The presence of diverse organic compounds and dyes in the wastewater affects various water quality parameters, including chemical oxygen demand (COD), biochemical oxygen demand (BOD), and total organic carbon (TOC).^4,5^ Human carelessness in releasing industrial wastes directly into water bodies leads to escalating levels of water contamination.^6^ Moreover, the widespread use of antibiotics in curing diseases causes large-scale pharmaceutical spills into waterways, endangering human health and the environment.^7^ However, mishandling pharmaceutical products, including human medicines and veterinary drugs, will significantly impact the environment.^8^ Pharmaceutical and personal care products are highly stable and resistant, hence posing various risks to human health, environmental safety, and aquatic systems. Some examples include antibiotics like tetracycline, meloxicam, ciprofloxacin, and ofloxacin.^9^ The primary sources of pesticides include agricultural run-off and urban landscaping activities. Further, water contamination by heavy metals is a major problem since they have the capability of causing serious adverse effects.^10^ In addition, the presence of heavy metals in the water resources can cause serious health consequences, including cancer and organ damage. Certain metals, like lead and mercury, may cause brain damage and autoimmune disorders.^11^ Moreover, their mutagenic, carcinogenic, and poisonous effects majorly impact the ecosystem.^12^ There have been several approaches devised for the treatment of wastewater, which are commonly classified into three types, which include biological, physical, and chemical processes. The wastewater may be subjected to biological treatment methods by using natural elements like bacteria, but biological processes have several disadvantages, including the cost and toxicity issues associated with them. Membrane filtration and adsorption techniques may prove to be highly effective, but there is no denying the fact that their efficiency is somewhat limited. However, an advanced oxidation process is considered highly promising because it helps to oxidize complex pollutants to harmless elements.^13^ If the dye molecules are broken down through the process of advanced oxidation methods, then there is a chance that all dye molecules will undergo complete mineralization. Among these, photocatalysis is one of the most efficient methods. Photocatalytic breakdown is a potential way to treat polluted water.^14^ Sustainable technology development is highly dependent on photocatalysts, which make use of light energy to carry out some important chemical transformations to enable energy production, storage, and wastewater treatment.^15^ Several kinds of metal oxide photocatalysts have been widely employed for photocatalytic purposes. Zirconium dioxide (ZrO_2_) is an n-type semiconductor material and performs effectively when used under UV light for photocatalytic processes. This material has a wide bandgap value (3.25–5.1 eV) and contains many oxygen vacancies on its surface. It has been enhanced by its high surface-to-volume ratio, whereas its mechanical properties are favourable for engineering applications. In addition, zirconium dioxide is cost-effective, eco-friendly, chemically stable, and non-hazardous. Its effectiveness is enhanced when combined with visible-light-active semiconductors.^16,17^

**Fig. 1:**
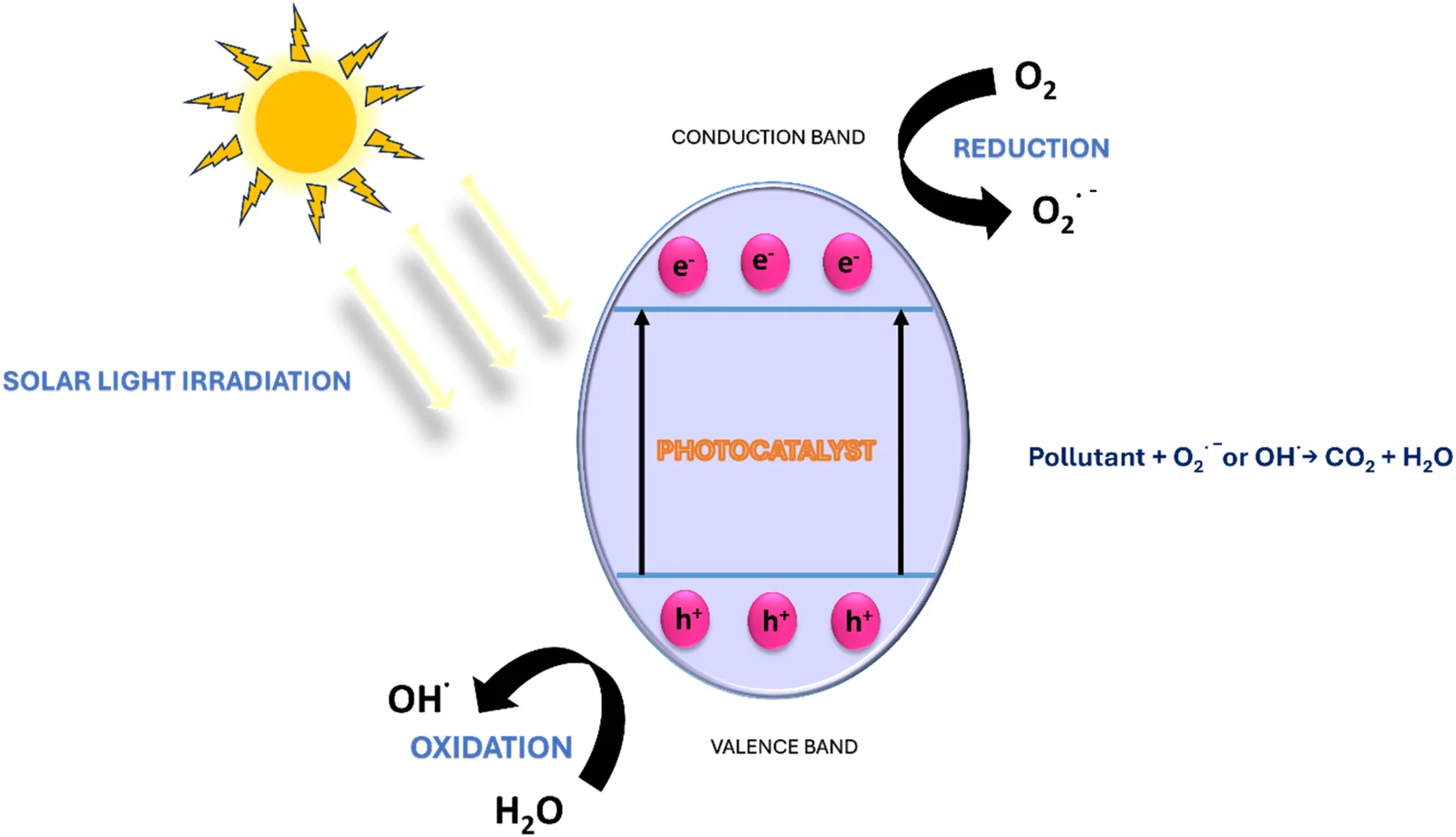
Photocatalytic degradation mechanism illustrating charge separation, radical formation, and pollutant decomposition.

Photocatalysis refers to a method by which light is employed to speed up a chemical reaction in the presence of a catalyst. Fig. 1 illustrates how, in the case where the photocatalyst absorbs light having an energy level equal to or higher than the bandgap of the semiconductor material, excitation of the electrons occurs by elevating the electrons from the valence band (VB) to the conduction band (CB), thus forming electron–hole pairs. The formation of electron–hole pairs requires proper separation to avoid recombination, which may lead to inefficiency in photocatalysis.^18^Metal oxide + *hυ* → e_CB_^−^ + h_VB_^+^h_VB_^+^ + H_2_O → OH˙ + H^+^ (oxidation)e_CB_^−^ + O_2_ → O_2_˙^−^ (reduction)O_2_˙^−^ + pollutant → H_2_O + CO_2_OH˙ + pollutant → degraded products

The electron–hole pairs migrate to the surface of the photocatalyst, where oxidation–reduction takes place between adsorbed organic substances, and this phenomenon is known as heterogeneous photocatalysis. It has been widely researched due to its relevance to environmental treatment and energy applications. Heterogeneous photocatalysis involves oxidation reactions occurring more often than reduction reactions in the presence of light energy. But electron–hole pairs tend to recombine, hence making the process inefficient.^19^ Photocatalysis is an advanced oxidation technique that holds great promise for the treatment of wastewater. This process is greatly affected by the characteristics of the materials used and operational parameters. It is crucial to comprehend these elements to maximize effectiveness and accomplish successful pollutant degradation.^20^

The use of such a technique can be highly efficient when it comes to contaminant destruction because of the production of reactive oxygen species. Reduction of waste and energy, as well as utilizing the source that will not be depleted in the future, like solar light, makes the process sustainable.^21^ The technology finds applications in diverse areas, ranging from water splitting to generate hydrogen, solar energy conversion, CO_2_ and N_2_ reduction, air and water purification, as well as potential biomedical applications. Photocatalysis provides a sustainable approach towards clean energy production, environmental protection, and the circular economy.^22^

The current developments regarding ZrO_2_-based photocatalysts under visible-light irradiation for water purification applications. It starts with an introduction to the current pollution crisis faced due to pharmaceuticals, pesticides, and heavy metals, as well as the limitations of conventional wastewater treatments. Fundamental concepts about photocatalysis and factors affecting its performance are briefly introduced, as well as the intrinsic nature, merits, and limitations of ZrO_2_ as a photocatalyst material.

Several methods have been proposed for engineering the large bandgap of ZrO_2_ for photocatalytic reactions under visible-light irradiation conditions. More emphasis has been put on the heterojunction design between ZrO_2_ and various metals, semiconductors, and carbonaceous compounds. Moreover, different synthesis methods, charge transfer processes, and ways to activate ZrO_2_ with visible light, along with the use of dopants and cocatalysts, have been reviewed in detail. Applications of ZrO_2_ heterojunction photocatalysts in degrading organic pollutants, inorganic species, and emerging contaminants are critically assessed along with current research issues. Earlier review articles give an overview of ZrO_2_ photocatalysis and deal with various aspects such as doping, defect engineering, morphology control, surface modification, and environmental application. This review mainly concentrates on heterojunction photocatalysts containing ZrO_2_ and establishes an understanding of their heterojunction type and band alignment with respect to interfacial charge transfer process, charge separation, reactive oxygen species formation, and photocatalytic performance by reviewing the existing experiments that have been utilized to identify Type-II, *Z*-scheme, and *S*-scheme mechanisms.

## 2. ZrO_2_ as photocatalyst

ZrO_2_ nanoparticles are being discussed more, since they have unique characteristics, including a high thermal expansion coefficient, high optical transparency, very strong mechanical stability, high corrosion resistance, and other potential uses for different purposes.^23^ They have unique redox and chemical stability over an extended pH scale, high surface area, flexibility, nontoxicity, and recyclability. There are three forms of pure zirconia, and these are cubic (c-ZrO_2_), tetragonal (t-ZrO_2_), and monoclinic (m-ZrO_2_) crystal phases, which profoundly impact the catalyst activities.^24^

Various intrinsic properties of zirconium dioxide prove it to be a very promising material for environmental and photocatalytic applications. First, the electronic properties are quite favourable, since in contrast to titanium dioxide and zinc oxide, its valence band potential energy (+3.8 eV) and conduction band potential energy (−1.1 eV) possess strong oxidizing and reducing powers, respectively, increasing the efficiency of the generation of free radicals and degradation of pollutants. However, there are two major disadvantages associated with ZrO_2_. They include the presence of electron–hole recombination rates and a wide bandgap (>4 eV), which hinder the photocatalytic activity of ZrO_2_ under visible light irradiation conditions.^25,26^ ZrO_2_ is more stable in alkaline and acidic conditions and can be produced in various shapes. It should be noted that ZrO_2_ demonstrates high chemical stability and the ability to form in various shapes. Still, due to the low efficiency associated with the mentioned factors, further research on enhancing its photocatalytic properties is necessary.^27^

The characteristics of the ZrO_2_ nanoparticles as catalysts and adsorbents make them a great choice for pollutant degradation. It is particularly exciting to note the ability of these nanoparticles to degrade dyes, especially where nanocomposites based on ceramics are concerned. One particular advantage of these nanocomposites lies in their ability to be reused over multiple cycles without losing their properties as adsorbents. Therefore, their commercial viability is assured as well.^28^ This material has a large bandgap, making it inefficient with respect to visible light. Modifications, including platinum doping and the addition of reduced graphene oxide, improve this process.^29^ ZrO_2_ acts as a very effective photocatalyst under UV-C light because of its wide bandgap. The best photocatalytic activity occurs when the material is annealed at temperatures ranging from 500 to 700 °C. Temperatures above 800 °C introduce interstitial oxygen sites, causing charge carriers to recombine effectively, hence poor photocatalytic activity. To increase the potential of zirconium dioxide, as well as make it versatile, doping and structural modifications are required.^30^

Due to its unique surface morphology, ZrO_2_ possesses some advantages as a photocatalyst. Large surface area, along with light scattering ability due to the porous nature of ZrO_2_, improves the degradation process. Oxygen vacancies play an important role, since they prevent the recombination process. With the help of doping, these oxygen vacancies can improve performance by increasing the absorbance in the visible light range. However, there are also some disadvantages of ZrO_2_ photocatalysts, like poor crystallinity, low porosity, less pore volume, and rapid charge recombination.^31^ But their economic viability and non-toxic nature make them ideal for environmental applications. Doping increases their ability to absorb light, charge separation, and stability of certain useful phases that increase photocatalytic efficiency. Moreover, doping helps to increase the surface area, which favours any reaction mechanism involving photocatalysis. The high recombination of electrons and holes resulting from the photochemical process on pristine ZrO_2_ reduces the stability of the charges, thus representing one of the challenges.^32^ Decreasing the bandgap improves light absorption and significantly enhances the photocatalytic efficiency.^33^

### 2.1 Strategies to enhance ZrO_2_ photocatalytic activity

Sahin *et al.* confirmed that, with control over critical parameters, the photocatalytic activity of ZrO_2_ thin films is essential for eliminating organic contaminants such as methylene blue. Notably, Zr acetate is much better than other sources of zirconium. Its degradation efficiency is 80%, compared with 16% for Zr nitrate and 54% for Zr chloride. The reason for these improvements lies in crystallinity, surface area, optical properties, and a reduced number of oxygen vacancies. The best annealing temperature is necessary to increase the efficiency of the process. There are several advantages of using ZrO_2_-based heterojunctions for photocatalytic purposes. In addition, ZrO_2_ provides the heterojunctions with stability and durability, and their peculiar topology allows for additional active sites and enlarged surface area for pollutant adsorption. However, there are challenges like a complex synthesis process and interface defects, which can hinder scaling up. Despite all of this, ZrO_2_ heterojunctions demonstrate higher photocatalytic activity due to efficient charge carrier separation, increased spectral responsivity, optimal band edge potential, increased surface area, and improved stability.^34^

In the case of visible light-driven photocatalysis of thiophene oxidation, pristine ZrO_2_ shows moderate catalytic properties on its own, as it has a photocatalytic efficiency and reaction rate constant of 38.2% and 0.0049 min^−1^, respectively. The only disadvantage of this catalyst is that there is a rapid recombination of charge carriers. ZrO_2_ nanocomposites show improved separation of charge carriers, decreased bandgap energy, and enhanced absorption of visible light. Thus, the photocatalytic efficiency increases greatly when combined with some semiconductors like CuO.^35^ The synergistic effect between CuO and ZrO_2_, CuO–ZrO_2_ nanocomposite shows great efficacy in removing cationic and anionic dyes (RB dye with degradation efficiency of 92.5% and BB dye with 76.4%). These compounds have a bandgap of 2.4 eV; they operate effectively under visible light. This ensures that they can be applied in case there is no UV light available. It possesses great chemical stability and reduced recombination of electrons and holes. In the experiments on recyclability, there was minimal degradation activity loss after a few cycles (about 12.69% for RB and 16.5% for BB dyes). In addition, CuO–ZrO_2_ nanocomposites are eco-friendly and therefore suitable for long-term application. Further research should be carried out for the commercialization process.^36^

Approaches that have been applied to improving the photocatalytic performance of ZrO_2_ include the addition of homogeneous nitrogen-doped oxides with ZrO_2_ nanoparticles, particularly MgTaO_6−*x*_N_*x*_. The presence of ZrO_2_ enhances the water reduction and oxidation reactions in the process of photocatalysis through the effective prevention of the formation of reduced tantalum states (Ta^4+^), which are the main centres of recombination affecting the efficiency of photocatalysis. Because the use of ZrO_2_ reduces the molar ratio between Ta^4+^/Ta^5+^, the modification approach involves not only inhibiting the formation of reduced tantalum but also having defect density in tantalum (oxy) nitride.^37^

Other catalyst materials, such as ZnO, MoS_2_, Bi_2_S_3_, and V_2_O_5_, can be used with ZrO_2_ to form composites. These may improve photocatalytic efficiency, electron transfer, and light absorption. Defect tuning, heterojunction engineering, and nanomaterial fabrication have the potential to boost the efficiency of the photocatalyst in addressing environmental challenges.^38^ Basahel *et al.* investigate the effect of the crystal form of ZrO_2_ on the photocatalytic oxidation of methyl orange. The monoclinic form exhibited the highest degradation efficiency among all the forms in 110 min, at a level of 99%. This remarkable achievement is credited to its abundance in hydroxyl groups and excellent crystallinity. The cubic form of ZrO_2_, despite being highly porous, exhibited lower efficacy.^39^

The efficiency of ZrO_2_ can be enhanced by altering some of its surface properties, electrical properties, and structure. For instance, N-doped graphene oxide encapsulated copper oxide and ZrO_2_ catalysts that inhibit charge recombination represent one of the ways through which nitrogen doping could significantly influence the performance of ZrO_2_ as a photocatalyst. Copper and ZrO_2_ are known to reduce the bandgap and hence increase the light absorption. Properties of active sites, which include the dual-interface architecture, control the catalytic activity.^40^ Another study shows that the full utilization of ZrO_2_ is possible by incorporating it within plasma electrolytic oxidation coatings and layered double hydroxide (LDH) materials. This technique will address the challenges of each material on its own, which include a lack of catalytic properties when using plasma electrolytic oxidation (PEO) coating alone and low structural stability associated with LDH materials. As a stable substrate with high surface area, the porous layer of PEO will enhance the stability and dispersion of LDH particles, preventing their aggregation. ZrO_2_ nanoparticles cause drastic changes in the surface morphology of the PEO coatings; therefore, they enhance surface area and reduce the bandgap.^41^ Degradation efficacy is highly dependent on the amount of ZrO_2_ nanoparticles used as a catalyst. An increase in the concentration of the catalyst from 10 to 25 mg in a 50 mL dye solution leads to a significant increase in the degradation rate. However, due to light scattering and shielding effects, high concentrations of the catalyst lead to particle aggregation, reducing the effective surface area and preventing the absorption of photons. Degradation rates are significantly affected by the concentration of the dye. Scavenger tests help to determine the reactive species responsible for deterioration. The pH level influences the structure, charging, and oxidation process, and it significantly affects the degradation of dyes using nanoparticles.^42^

## 3. Comparative analysis of heterojunction formation

The photocatalytic reaction is initiated by the movement of electrons from the valence band to the conduction band of the semiconductor material, thereby creating an electron vacancy in the valence band. Orientation, built-in potential field and energy barrier, band position, and interface conditions are among the various factors influencing heterojunction behaviour. Some different kinds of heterojunctions include Schottky barrier heterojunction, p–n and non-p–n semiconductor heterojunction, van der Waals heterojunction, Facet heterojunction, Type-I or straddling gap, Type II or straggling gap, *Z*-scheme, *S*-scheme, and Type III heterojunctions.^43,44^

The concept of heterojunctions in photocatalysis refers to the integration of two or more dissimilar semiconductor materials to form an interfacial contact, which facilitates efficient charge separation and transfer, thereby enhancing photocatalytic activity.^45^ In a p–n semiconductor, if there is a contact between an n-type and a p-type semiconductor, then a space charge region will form as a result of the movement of charges in opposite directions to form a junction. The Schottky junction, characterized by reduced recombination reactions and separation of charges, can be seen between a semiconductor and a metal. In conventional *Z*-schemes, the major problems include the reversibility of redox mediators and applicability in a liquid medium. For solid-state *Z*-schemes, the electron mediators include materials like graphene, carbon nanotubes, or noble metals. The advantage of the *S*-scheme lies in the separation of charges and sustaining the high redox potential of charges.^46^

Heterojunctions are a kind of photocatalyst that combines the benefits of several components to overcome the drawbacks of single semiconductor photocatalysts. They improve charge separation, charge transfer, and the thermodynamic and kinetic aspects of photocatalytic processes. Electrons can move from the conduction band of one semiconductor to the conduction band of the other in Type I heterojunctions because the bandgap of one semiconductor is contained within the forbidden band of the other semiconductor.^47^ Some approaches to improve Type I heterojunctions involve improving the efficiency of interface engineering by using carbon dots at the core–shell interface, which makes it easier for the electrons to move from one part of the heterojunction to another, resulting in greater photocurrent, decreased impedance, and prolonged lifetime of the carriers. It is thus evident that enhanced charge separation can be achieved in Type I structures, which are less efficient due to rapid recombination and fewer redox properties. However, when Type I heterojunctions are improved through good interface mediators and particular designs such as hollow spheres, these can yield results comparable to Type II and *Z*-schemes.^48^

While Type II (staggered band) heterojunctions were initially devised in order to deal with the problem of rapid electron–hole recombination in semiconductors, they are not considered very efficient as compared to the advanced *Z*-schemes and *S*-schemes. A Type II heterojunction involves two semiconductors whose energy bands are staggered. Consequently, it becomes possible for electrons to transfer from the higher energy band of one semiconductor to the lower CB of another semiconductor. The advantages of Type II heterojunction catalysts include efficient carrier separation, as well as enhanced light absorption and large surface areas that facilitate improved transport mechanisms.^49^

**Fig. 2:**
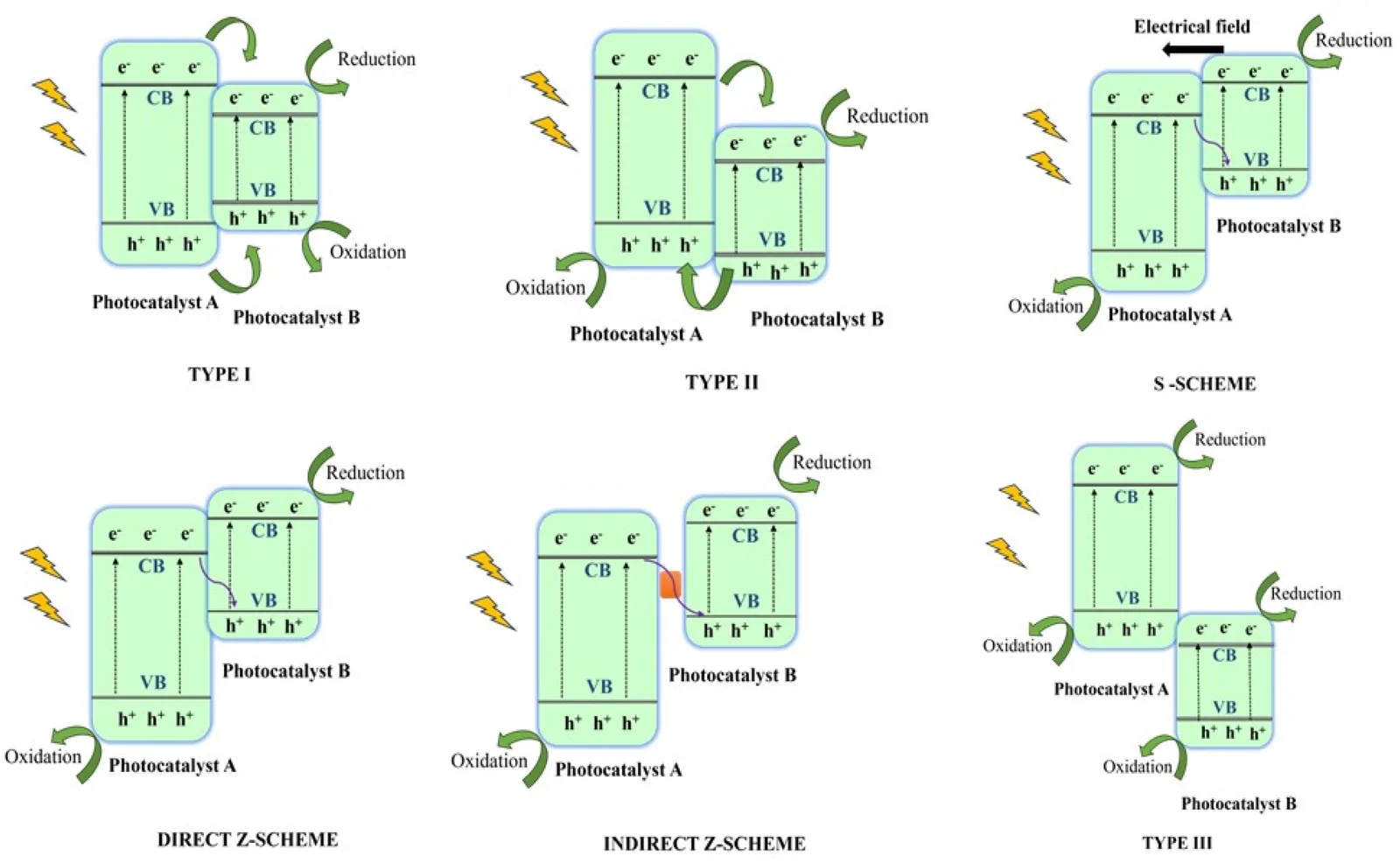
Schematic illustration of different types of heterojunctions.

*Z*-scheme heterojunction photocatalysis was developed to address two main issues that arise when using a single semiconductor in photodegradation reactions, namely, insufficient utilization of visible light and unstable redox pairs. Two semiconductors are combined such that while one provides powerful oxidizers (holes), the other produces strong reductants (electrons). As opposed to the conventional heterojunction Type-II structures, the *Z*-scheme heterojunctions maintain good redox power while improving charge carrier separation through spatial separation of the electrons that are highly reducing as well as the oxidizing holes within two photocatalysts. The photocatalysts I and II are linked in this arrangement, making possible the recombination of the photogenerated electrons. The *Z*-scheme can be classified into two types, namely, the direct *Z*-scheme, where solid contact occurs between the photocatalysts, hence eliminating mediators and showing higher efficiencies in applications such as organic degradation and CO_2_ reduction and the indirect *Z*-scheme that employs a redox couple in the charge carrier recombination process, leading to backward reactions. Fig. 2 represents the types of heterojunctions. However, there are several limitations to consider, such as the requirement for expensive material components for solid-state *Z*-schemes, interface recombination issues for direct *Z*-schemes, and reverse reaction effects for indirect *Z*-schemes. Despite all of that, *Z*-schemes appear to be a good choice, but further advancements are required.^50,51^

*S*-scheme heterojunctions are a newer class of photocatalysts designed to fix key conceptual and practical flaws in Type-II and *Z*-scheme designs, while retaining strong redox power and good charge separation. They are built around oxidation and reduction photocatalysts and are carefully engineered with interfacial electrical fields. The use of an oxidation photocatalyst together with a reduction photocatalyst leads to the creation of an *S*-scheme heterojunction that enhances redox potential and charge separation. When both are brought into contact, the electrons from the reduction photocatalyst will shift to the oxidation photocatalyst, thus developing an internal electrical field and band bending. The synergistic combination of a catalyst that can do both redox reactions or integrating two individual catalysts capable of oxidation and reduction at the same time leads to the formation of *S*-scheme heterostructures. But the problems related to interfacial physics, mechanism identification, and increased efficiency persist. The *S*-scheme heterojunction suffers from many practical problems, but in general, they do seem to hold some potential.^52,53^

It should be noted that the *S*-scheme heterojunction operates on the basis of an internal electric field and is different from Type-II heterojunctions, where there is a direct electron transfer between conduction or valence bands. The efficiency of photocatalytic reactions depends on the charge-state dynamics and interactions of carriers and improves properties such as light harvesting. But there are difficulties in classifying heterojunctions into *S*-scheme, which leads to possible misconceptions. Enhanced redox capabilities, efficient charge separation, and stability are some of the pros of the *S*-scheme, while complexity, challenges in characterization, and interfacing issues are some of its cons. Future research areas may include standardized approaches, modelling, development of novel materials, scaling for commercial applications, and understanding the interfacial dynamics.^54^

The uncertainties regarding alignment and the theoretical criteria complicate the mechanism, particularly in the case of Type-II transfer. This clearly indicates how the orientation of electric fields, hindering the process of Type-II transfer, can become an impossible process if the Fermi level of photocatalyst 1 is higher with a more negative CB. The position of the Fermi level is crucial as it determines the type of transfer. According to the findings from research, it is necessary to retain a driving force for *S*-scheme transfer, but the experimental data reveals that contact resistance and interface flaws often pin Fermi levels, undermining the expected dynamic equilibrium required for efficient operation. Dual *S*-scheme heterojunctions with several photocatalysts, tuning of Fermi level gradients, and inclusion of chemical bonds at the interface are some methods to overcome these issues. Currently, there are challenges involving the complexity of transfer pathways and factors other than interfacial electrical fields, such as contact resistance and diffusion. To understand the dynamics of carriers, there is a need for advancements that will help to address current research gaps and pave the way for more future developments in *S*-scheme heterojunction applications.^55^

Due to the highly staggered nature of the Type III heterojunction, wherein the conduction band of one semiconductor is placed below the valence band of the other semiconductor, there is a lack of continuity in the gap, which limits the effective migration and separation of photo-generated electron–holes.^56^ There exists a unique category of semiconductor junctions called Type III heterojunctions, characterized by a “broken-gap” alignment of bands, resulting in poor charge separation. Due to the fact that they do not overlap, the Type III heterojunction requires a higher driving pressure compared to other heterojunctions like Type I/II/Z/S. However, Type III heterojunctions exhibit certain limitations, such as poor charge separation and the need for additional conductive material to make them useful, among others.^57^

## 4. Visible-light driven ZrO_2_-based photocatalysts

The ability to achieve the separation and transportation of electron and hole pairs generated during the photoexcitation process becomes possible when a heterojunction, especially a *Z*-scheme and *S*-scheme heterojunction, is used. The reduction in ZrO_2_ bandgap is accomplished through doping or creating a heterojunction, allowing the material to have the ability to absorb different wavelengths of light, including visible light. In order to improve the performance of photocatalysis, certain modifications can be carried out, such as heterojunctions. Although Type-I and Type-II heterojunctions (for example, ZrO_2_/ZnO and ZrO_2_/PPy) increase the rate of pollutant degradation in both UV and visible light, they suffer from a charge accumulation problem. *S*-scheme heterojunctions and *Z*-scheme systems. Such as g-C_3_N_4_/ZrO_2_, improve redox ability and charge carrier separation. In addition to this, surface area and morphology variations through methods such as hydrothermal and ultrasonication methods improve photocatalytic activity.^58^

Adaileh *et al.* research employs a Cu–ZnO/ZrO_2_-based polyacrylonitrile polymer composite heterojunction for the degradation of contaminants under visible light conditions. The mechanism for this process can be explained with the aid of the pseudo-second order rate equation, which provides a better estimate of experimental results compared to the pseudo-first order reaction. The lowering of the bandgap energy to 2.38 eV and thereby ensuring efficient energy absorption from 200–500 nm allows this heterojunction to efficiently degrade pollutants under visible light conditions.^59^ The formation of heterojunctions with other oxides through reduction of charge carrier recombination is another way of enhancing the photocatalytic properties of ZrO_2_. An example is the formation of heterojunctions with Fe_2_O_3_, whose semiconductor properties make it possess a low bandgap, making the overall photocatalytic properties better as a result of increasing its surface area and ability to absorb visible light. The low band gaps are the reason why the dopants, such as Fe_2_O_3_, are more efficient and can therefore degrade pollutants such as Congo Red. The catalysts are also assisted by biopolymer cocatalysts like nanocellulose.^60^

The g-C_3_N_4_/ZrO_2−*x*_ nanotube composites and ZrO_2_/g-C_3_N_4_ heterojunctions both demonstrate enhanced visible-light-driven photocatalytic activity due to the formation of heterojunction interfaces between ZrO_2_ and g-C_3_N_4_. In the g-C_3_N_4_/ZrO_2−*x*_ system, the presence of oxygen vacancies reduces the bandgap to 2.9 eV, enhances visible-light absorption, improves charge transport, and increases the surface area, resulting in up to 90% tetracycline degradation within 1 hour under visible light irradiation. The improved performance is further facilitated by a *Z*-scheme charge transfer pathway that preserves strong redox potentials. Similarly, ZrO_2_/g-C_3_N_4_ heterojunctions exhibit superior photocatalytic degradation of organic dyes, including Rhodamine B, Methyl Orange, and Acid Orange II, owing to enhanced visible-light absorption and efficient charge separation through the *S*-scheme mechanism. While both systems benefit from the incorporation of g-C_3_N_4_ and improved charge carrier dynamics, the g-C_3_N_4_/ZrO_2−*x*_ composite relies on oxygen vacancy-induced bandgap narrowing and a *Z*-scheme pathway, whereas the ZrO_2_/g-C_3_N_4_ heterojunction achieves enhanced photocatalytic efficiency through an *S*-scheme charge transfer mechanism and improved electrochemical properties.^61,62^ These findings suggest that both *Z*-scheme and *S*-scheme architectures are effective strategies for enhancing the visible-light photocatalytic performance of ZrO_2_ heterojunctions.

Aljuwayid *et al.* investigated that, by overcoming the rapid recombination of photoexcited charge carriers, the *S*-scheme approach improves the photoactivity of the material. The photocatalytic ability of the nanostructure made up of C_3_N_4_ and ZrO_2_ is better than that of other materials due to enhanced charge transfer in the interface of C_3_N_4_ and ZrO_2_. Higher photocatalytic activity is determined by a low recombination rate. Upon being exposed to simulated sunlight for 140 minutes, the nanostructure has the highest photocatalytic activity for degrading mixed dyes at 97% efficiency. Furthermore, it has larger pores and a high surface area, thus enhancing the adsorption of mixed dyes.^63^

As a consequence of increased light absorption and charge carrier separation, ZrO_2_/heteropoly blue heterojunction photocatalysts can be considered an effective means of improving photocatalytic efficiency. Being a type of polyoxometalate, heteropoly blue possesses distinct electrical properties and band gap energy values. They also exhibit higher light absorption properties, making them excellent photocatalysts. ZrO_2_/HPB heterojunction photocatalysts are produced using a two-step method that includes hydrothermal and precipitation reactions. The heterojunction creates the smallest semicircular arc and photocurrent because it promotes directed charge migration and decreases the rate of recombination. The optimization of Type-II ZrO_2_/HPB heterojunction formation occurs by varying the calcination temperature.^64^

El-Hosainy *et al.* proposed a study to increase the sensitivity to visible light and ensure efficient charge carrier separation. The NiS@ZrO_2_ heterojunction improves visible light absorption, thereby increasing photocatalytic efficiency significantly. The reduction in bandgap energy and phase shift in ZrO_2_ towards higher wavelengths owing to the addition of NiS renders the material applicable under visible light irradiation. To maintain a strong redox potential, an *S*-scheme charge transfer process is proposed by this study, outperforming conventional Type-II schemes. Moreover, the presence of nanocomposites with high surface area allows the creation of extra hydroxyl groups and the movement of CIP molecules to reaction sites. The optimized 2 wt% NiS@ZrO_2_ achieved complete degradation within 45 min.^65^

The Ag/ZrO_2_/g-C_3_N_4_ and Ag/ZrO_2_/TCN plasmonic heterojunction photocatalysts both exhibits enhanced visible-light driven photocatalytic performance owing to the incorporation of Ag nanoparticles and the formation of efficient heterojunction interfaces. In the Ag/ZrO_2_/g-C_3_N_4_ system, the dual heterojunction structure promotes visible light absorption and effective charge carrier separation, while the unique sandwich architecture facilitates charge transfer between g-C_3_N_4_ and ZrO_2_. The Ag nanoparticles serve as electron-conduction mediators, further improving charge transport. Similarly, Ag/ZrO_2_/TCN demonstrates remarkable stability and strong photocatalytic degradation capability under visible light irradiation. However, despite their enhanced photocatalytic activity, Ag/ZrO_2_/TCN photocatalysts face challenges such as particle agglomeration, gradual efficiency loss after repeated cycles, and depletion of noble metals.^66,67^ These findings indicate that although Ag-based ZrO_2_ heterojunction systems effectively improve visible-light photocatalysis through enhanced charge separation and transfer, further efforts are required to address stability and durability issues for long-term applications.

Even though ZrO_2_ possesses excellent redox properties and is an extremely flexible material, it has a relatively poor efficiency rate when exposed to visible light because of its wide bandgap. Consequently, the ZrO_2_/g-C_3_N_4_ nanocomposite showed improved photocatalytic behaviour, breaking down the pollutants when exposed to solar light. In addition, the nanocomposite reduces the wide bandgap from 3.6 eV to 2.8 eV.^81^

The heterojunction structure of the material named zeolite@A-ZrO_2_, which is made up of zeolite and amorphous ZrO_2_, has been developed as a novel means of enhancing the degradation performance of Rhodamine B, the large surface area, and the stability of zeolite with surface defects of amorphous ZrO_2_. Due to the high level of stability and reusability of the zeolite@A-ZrO_2_ catalyst, it is very suitable for use in dye wastewater treatment systems that will last for a long time. In five consecutive cycles, it exhibits 98.43% degradation efficiency, implying minimal loss in terms of performance. The favourable features of zeolites in relation to their function as excellent carriers, as well as the interfacial contacts developed as a result of the amorphous ZrO_2_ layers due to the efficiency of electron–hole separation, are some of the reasons that make it stable. Its functionality in environmental cleanup.^82^

## 5. Synthesis method for the ZrO_2_-based nanocomposites

### 5.1 Hydrothermal method

**Fig. 3:**
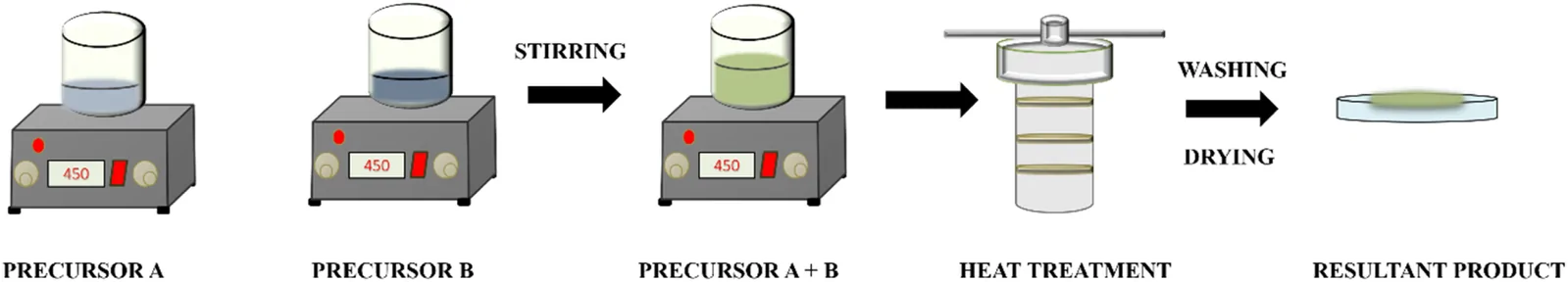
Schematic steps involved in the hydrothermal synthesis route.

Silver-doped ZrO_2_ was synthesized by Nova *et al.* using the hydrothermal process; Fig. 3 shows the general hydrothermal synthesis method. ZrO_2_ nanoparticles were determined to have sizes from 5 to 8 nm, which resulted in the formation of a tetragonal crystal structure confirmed by Raman and XRD analysis. With a wavelength of 467 nm, Ag@ZrO_2_ nanocomposites exhibited greater photocatalytic activity, being able to decompose more than 98% of Rhodamine B dye in 60 min as compared to 140 min for pure ZrO_2_ nanoparticles. This activity is due to a metal/semiconductor heterojunction that provides better charge separation and reduction of electron–hole pair recombination. However, problems concerning the shape of nanoparticles and their agglomeration persist. Despite that, the synthesis procedure is known for its simplicity, cost-effectiveness, and the antimicrobial properties of Ag@ZrO_2_ nanocomposites.^83^

Synthesis of ZrO_2_-based heterojunction photocatalysts using an ultrasonic hydrothermal approach is reported by Jiang *et al.* The material is synthesised by mixing g-C_3_N_4_ and Ni–ZrO_2_ in methanol as the solvent, followed by drying and calcination. *S*-scheme charge transfer model promotes efficient separation of charge carriers and maintains the photo-induced redox pairs. Reduced redox capacity and inadequate electron transport are the limitations of the Type-II heterojunctions. While *Z*-scheme mechanisms are designed to preserve redox carriers and speed up their separation, they still have limitations such as side reactions and light shielding. The *S*-scheme mechanisms make use of an internal electric field to enhance redox activity. This optimized composite material (40% NZO/CN) shows better performance compared to both pristine g-C_3_N_4_ and Ni–ZrO_2_ in terms of degradation performance. The ideal composition shows outstanding performance in tetracycline hydrochloride degradation up to 96.8%. This condition is under 0.02 g catalyst loading and pH value of 5.^84^

### 5.2 Sol–gel method

Importance of using a one-step sol–gel process and supercritical drying technique for the synthesis of ZrO_2_ aerogels. The procedure guarantees controlled solvent removal, limits structural contraction, and yields aerogels with large surface areas, like 174 m^2^ g^−1^ for ZrO_2_ calcined at 400 °C. Monoclinic ZrO_2_ calcined at 800 °C is equally active as ZrO_2_ 400 °C, despite having a lower surface area, due to the creation of oxygen vacancy sites on the surface at high temperatures. Oxygen vacancy sites act as electron traps, leading to the production of oxidizing radicals, as evidenced by photoluminescence and Raman spectroscopic studies.^85^

Kumari *et al.* synthesised the mixed phase of TiO_2_–ZrO_2_ nanocomposites through the sol–gel and solution combustion approaches. In order to synthesize ZrO_2_, zirconium(vi) oxynitrate hydrate was employed using the solution combustion process. Eosin yellow dye breaking down by 87.7% within 60 min under visible light, indicates the effectiveness of the mixed composite in the photocatalytic activity as compared to TiO_2_ and ZrO_2_. Superoxide and hydroxyl radical which leads to decomposition of organic compounds into CO_2_ and H_2_O results from enhanced charge separation and electron–hole recombination.^86^

Jingjun *et al.* study explores the application of zinc oxide, a semiconductor with excellent optical and photocatalytic properties, along with zirconium oxide, well known for its thermal stability and chemical inertness, to create nanocomposites through the sol–gel process, owing to its simplicity and low cost. The synergistic effects, such as a lower bandgap, which allows efficient absorption of visible light and generates more charge carriers, the combined materials having higher photocatalytic efficiency. It is possible to identify both the hexagonal and tetragonal phases using XRD, with a crystallite size of 18–19 nm. The remarkable efficacy of ZnO–ZrO_2_ nanocomposites in degrading organic dyes up to 96.89% of methylene blue under visible light.^87^

### 5.3 Co-precipitation method

In the case of the co-precipitation technique, the process is based on mixing both the metals, introducing a base to obtain a mixed compound containing ZnO/ZrO_2_ in form of oxides or hydroxides, ageing, and drying to produce a core–shell material consisting of amorphous Zr (4.9 wt% being optimal) coated over a wurtzite ZnO structure, providing excellent results in terms of Rhodamine B degradation (9 ppm) using visible light 220 W. Major parameters considered are exact composition ratio (1–9.3 wt% ZrO_2_), addition of CTAB surfactant, and oxygen vacancy generation.^88^

**Fig. 4:**
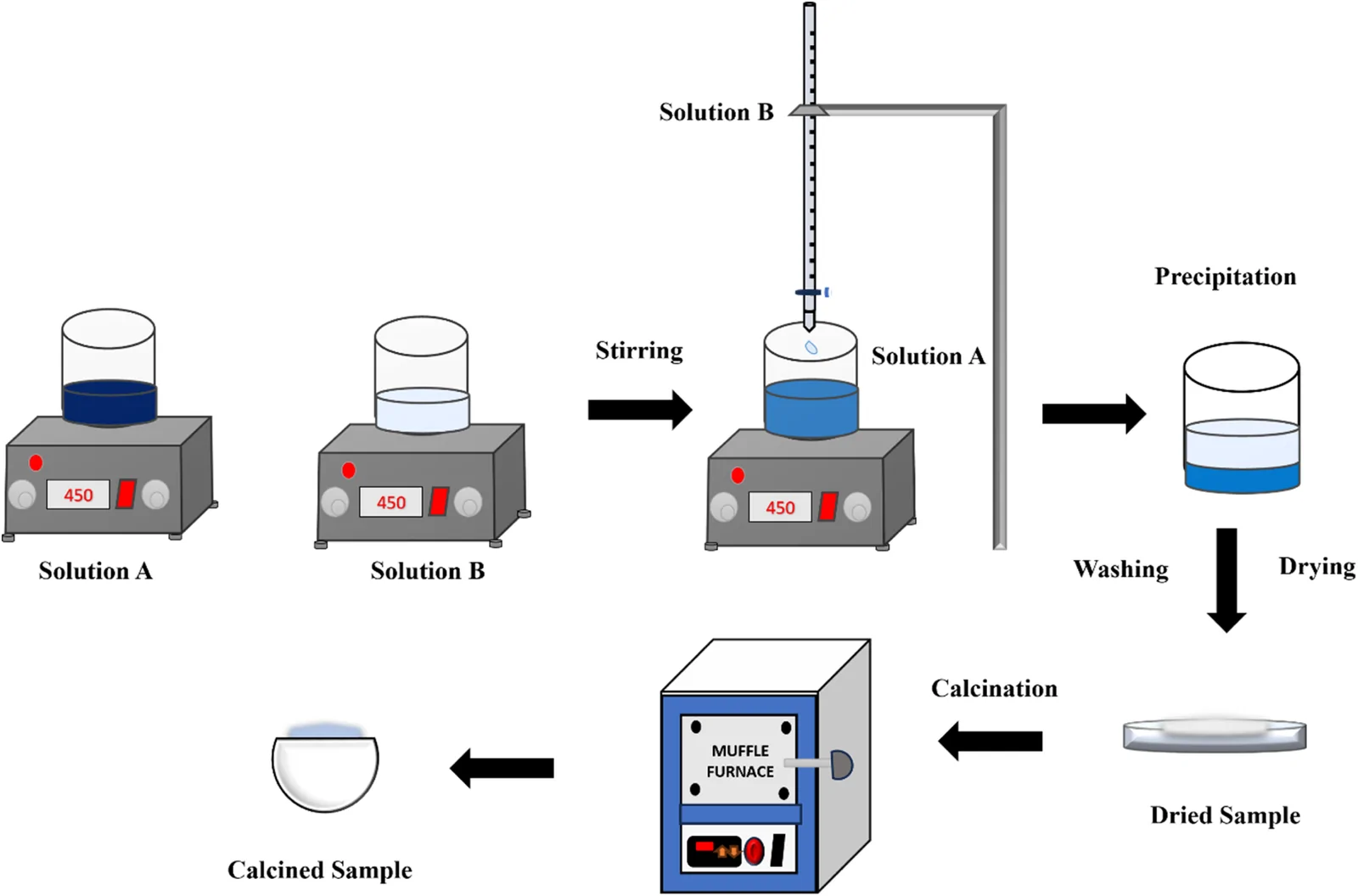
Illustration of the typical co-precipitation process used for material synthesis.

Bagri *et al.* synthesized the nanocomposites Bi_2_O_3_–ZrO_2_, which have increased characteristics, including a higher surface area (85.94 m^2^ g^−1^) and mesoporosity, favourable for both adsorption and photocatalysis, and can be synthesized using equimolar co-precipitation of Bi and Zr salts at pH 7. Despite blocking high redox potentials, the 2.91 eV bandgap allows efficient charge separation. The optimization of the co-precipitation method is constrained by the evaluation of only one sample and calcination conditions due to its scalable and low-cost preparation method. The optimal pH value for catalytic reactions is neutral, and when dealing with a mixture of pollutants, the reaction rate depends on the composition of the mixture. Increase of catalysts dose significantly enhances the clearance rates. While the nanocomposite demonstrates satisfactory reusability, the number of active sites becomes fewer during the course of six cycles.^89^ The typical co-precipitation illustration is shown in Fig. 4.

### 5.4 Green synthesis of ZrO_2_ nanoparticles

An extract of *Azadirachta indica* leaves is included in the process of reducing metal ions in the preparation of ZrO_2_ nanoparticles *via* the method of biosynthesis. The calculated bandgap value of it is significantly lower compared to that of bulk zirconium (>5 eV), overcoming limitations and enhancing its photocatalytic activities as well as its effectiveness for the degradation of methylene blue by 99.6%. Furthermore, they can show their antibacterial effect on Gram-negative bacteria.^90^ Other than giving an environmental approach in the creation of antibacterial nanoparticles and photocatalysts, the green synthesis technique of utilizing gum *Moringa oleifera* to produce TiO_2_/ZrO_2_ nanoparticles proves to be effective through their ability to degrade pollutants like Methylene Blue and Rhodamine B by 96.3% and 94.5%, respectively.^91^ Extract of *Passiflora edulis* extract was used to synthesize the ZrO_2_ that are made in spherical shape and of small sizes. They belong to the tetragonal phase and possess an average crystalline size of about 7 to 11 nm, respectively. They can also demonstrate increased biological activity and 99% degradation of Rhodamine B.^92^

Haq *et al.* used the leaves of the rubber tree as reducing and capping agents in the green chemistry-based formation of the ZnO–ZrO_2_ heterojunction. The photocatalytic degradation of Rhodamine 6G was considered, leading to a constant degradation rate of 0.0615 min^−1^ and 97.3% of dye degradation in 55 min. These results demonstrate superior properties compared to those of ZnO. This activity is facilitated by a property of the material, where the electrons of both materials are stimulated through the absorption of sunlight, and an electron transfer occurs from ZrO_2_ to ZnO.^93^

Shinde *et al.*, produced the g-C_3_N_4_–ZrO_2_ nanocomposite. Initially, *Ficus benghalensis* leaf extract was used as a stabilizing and reducing agent during the biosynthesis of ZrO_2_ nanoparticles. Coupling ZrO_2_ with g-C_3_N_4_ reduces the bandgap from 4.9 eV to about 3.4 eV and extends light absorption into longer wavelengths than g-C_3_N_4_ alone. The nanocomposites also show a large surface area, providing more active sites for pollutant adsorption and degradation. Optimal operational parameters include a catalyst loading of 150 mg per 100 mL of the MB solution and pH 7, where electrostatic attraction between negatively charged composite surfaces and cationic MB molecules maximizes degradation.^94^

*Butea monosperma* leaf powder was used as a natural fuel in a green combustion process to create the ZnO/ZrO_2_ heterojunction nanocomposite. Zinc nitrate hexahydrate and zirconyl nitrate hydrate were used as metal precursors. ZnO/ZrO_2_ nanocomposites were created by heating the mixture to 500 °C for 10 min. Nanoparticle production was aided by the hydroxyl group and phytochemicals in the leaf acting as reducing agents. The resultant nanocomposite demonstrated remarkable photocatalytic efficiency, breaking down 99% of Methylene Blue in 150 minutes. At pH 12, a catalyst dosage of 30 mg produced 98.3% degradation. After 150 min, the nanocomposite also demonstrated dual functionality by reducing Cr(vi) to Cr(iii) by 62%. The ZnO/ZrO_2_ catalyst also demonstrated outstanding stability and reusability, suggesting a great deal of promise for environmental remediation applications.^95^

Banana peel extract, which is rich in antioxidants, can be used to create reduced graphene oxide/zirconium oxide (rGO/ZrO_2_) nanocomposites in an eco-friendly manner. The resulting rGO-ZrO_2_ nanocomposites show improved charge carrier production and increased photocatalytic activity when compared to individual components because of the creation of a heterojunction. Because of its stability and reusability, this biogenic composite retains significant removal efficiency for Rhodamine B (80%), Crystal Violet (83%), and ciprofloxacin (88%) after five cycles of usage. Degradation efficiency is influenced by the ideal catalyst dosage. CIP degradation increased from 38.2% to 93.1% with an increase from 10 mg to 40 mg. Degradation is also influenced by pH levels, with neutral solutions promoting optimal photocatalytic activity.^96^

## 6. ZrO_2_-based ternary nanocomposites

Jayasinghe *et al.* examined TiO_2_, along with reduced graphene oxide in the form of rGO/TiO_2_/ZrO_2_, which shows an increase in photocatalytic degradation as a result of enhanced material properties. Mixed oxides reduce the bandgap energy for visible light activation with high efficiency. In addition, TiO_2_/ZrO_2_ increases electron transfer with phase stabilization. Finally, rGO acts as an electron carrier to separate charges and minimize recombination, resulting in an increased formation of reactive oxygen species.^97^Table 1 shows various ZrO_2_ ternary-based nanocomposites.

**Table 1:** ZrO_2_-based ternary nanocomposites

Ternary nanocomposite	Bandgap	Light radiation source	Pollutant	Time and degradation efficiency	References
Cu_4_O_3_/ZrO_2_/TiO_2_	2.91 eV	Solar light irradiation	Everzol yellow 3RS (EY-3RS)	91.41% in 100 min	68
NiFe_2_O_4_/TiO_2_/ZrO_2_	3.03 eV	Visible light irradiation	Alizarin Red S dye	100% in 40 min	69
ZrO_2_/CaCr_2_O_4_/BiOIO_3_	2.5 eV	Visible light irradiation	Cefixime and doxycycline	99.8% (CFX) in 340 min and 98.9% (DOX) in 220 min	70
NiS/ZrO_2_/CdS	—	Sunlight irradiation	Tetracycline	97.05% in 120 min	71
TiO_2_/ZrO_2_/SiO_2_	—	Sunlight irradiation	Reactive Red 120 (RR120) dye	96.2% in 75 min	72
ZrO_2_/Fe_2_O_3_/RGO	1.70 eV	Visible light irradiation	Congo Red and acetophenone	98.43% in 60 min (CR) and 97.76% in 80 min (AP)	73
ZrO_2_/Ag@TiO_2_	2.83 eV	Visible light irradiation	Azithromycin	90.93% in 8 h	74
ZrO_2_/TiO_2_/Fe_3_O_4_	—	UV light source	Naproxen	100% in 90 min	75
Fe_3_O_4_@m-ZrO_2_@Ag	—	UV light source	Rhodamine 6G, Cr(vi)	98.6% in 60 min and 100% in 30 min	76
ZrO_2_/NiO/RGO	1.75 eV	Visible light irradiation	Methylene Blue and Rhodamine B	100% (MB) and 99.9% (RhB) in 60 min	77
Polyaniline/phosphotungstic@ZrO_2_	1.35 eV	Visible light irradiation	Methylene Blue, *p*-nitrophenol, 2,4-dichlorophenoxyacetic acid	98% (MB) in 105 min, 99% (PNP) in 15 min, and 92% (2,4-D) in 600 min	78
ZrO_2_/CdZrO_3_/S_*xy*_	2 eV	Sunlight light irradiation	Crystal violet	95% in 30 min	79
rGO/ZrO_2_/Ag_3_PO_4_	2.3 eV	UV light irradiation	4-Nitrophenol	97% in 90 min	80

Omar *et al.* constructed the NiS/ZrO_2_/CdS dual *Z*-scheme photocatalyst using coprecipitation and hydrothermal methods. When comparing the results of emission of the composite photocatalyst with those of binary CdS/ZrO_2_ and NiS/ZrO_2_, a significant decrease in the emission intensities is observed, which means an improved behaviour of separation of the electrons from holes. This new process facilitates more light absorption and charge carrier separation compared with the other binary systems. The importance of this process in facilitating the decomposition of various antibiotics. Due to their effectiveness in light absorption and separation of charge carriers, these photocatalysts degrade various organic compounds efficiently.^71^

Saira *et al.* studied under the influence of solar spectrum radiation; the ternary Cu_4_O_3_/ZrO_2_/TiO_2_ nanocomposites degraded 91.41% of the initial concentration of 40 ppm EY-3RS in 100 minutes. Obviously, this value is much higher than that of pure TiO_2_ (81.48%) and the binary Cu_4_O_3_/ZrO_2_ (86.65%). Due to this, the exploitation of a large variety of wavelengths in the visible light region is possible because of the small optical bandgap of the ternary nanocomposites, which is 2.91 eV. This is certainly a significant progress compared to pure TiO_2_ with its larger optical bandgap of 3.42 eV. It means the use of solar radiation energy is much more effective in terms of photocatalytic organic compound degradation. Such a high steady-state concentration of the active species results from the combination of all these oxides, where ZrO_2_ provides the electron transfer, Cu_4_O_3_ delivers high-energy holes, and TiO_2_ serves as a substrate and electron mediator.^68^ Although both experiments indicate that the incorporation of ZrO_2_ in the ternary heterojunction structure that improves the photocatalytic performance, the key strengths of each lie in different interfacial processes. The Cu_4_O_3_/ZrO_2_/TiO_2_ system, for instance, enhances the utilization of solar energy by reducing the bandgap and utilizing complementing roles of the constituent oxides; the NiS/ZrO_2_/CdS mainly relies on charge separation in a *Z*-scheme.

Tavakoli-Azar *et al.* created a ternary nanocomposite, specifically ZrO_2_–CdZrO_3_–S_*xy*_, with sulfur added to improve the photocatalytic activity by a two-stage hydrothermal method followed by solid-state reaction. This approach enables more control over material properties and the production of the desired nanocomposite structure. Under natural sunlight, the ZrO_2_–CdZrO_3_–S_20_ nanocomposites showed the maximum degradation efficiency of crystal violet dye, achieving 95% degradation in the first half hour. By lowering the bandgap energy, sulfur was added, increasing the photocatalytic activity. According to the study, reactive species such as superoxide and hydroxyl radicals are essential to the degradation process.^79^

By using ultrasonication, an MgO@ZrO_2_@g-C_3_N_4_ nanocomposite was synthesized by Rasha to degrade Alizarin Red dye, with a 92% photodegradation efficiency. While MgO and ZrO_2_ produce heterojunctions that reduce their large bandgap and improve visible light absorption, charge separation, and redox activity. ZrO_2_'s oxygen vacancies lessen electron–hole recombination and promote charge transfer. The primary reactive species in the degradation pathway are holes and hydroxyl radicals. The composite is positioned by the study as a safe and affordable photocatalyst for wastewater treatment.^98^

Supriya *et al.* found that the enhancement of electron transportation, the combined effect of NiFe_2_O_4_, TiO_2_, and ZrO_2_, reduces charge recombination, thus boosting the photoactivity of the ternary compound. ZrO_2_ inhibits photo-corrosion and enhances the physical stability and durability of the ternary compound. The magnetic properties of NiFe_2_O_4_ allow it to be recovered and separated from the solution through the application of external magnets. As a catalyst, it decomposes dyes by 100% in 40 min when subjected to visible light. It also makes the ternary compound environmentally sustainable and reusable.^69^ The study indicates that enhancement of the use of light and charge separation leads to higher degradation of pollutants in ternary ZrO_2_-based photocatalysts. Electron–hole pair separation through the introduction of sulfur, creation of heterojunctions, and oxygen vacancy generation effectively boosts the photocatalytic efficiency. Moreover, the practical utility of the system is proved by its improved stability and magnetic recyclability of the NiFe_2_O_4_/TiO_2_/ZrO_2_ system.

## 7. Characterization techniques

**Fig. 5:**
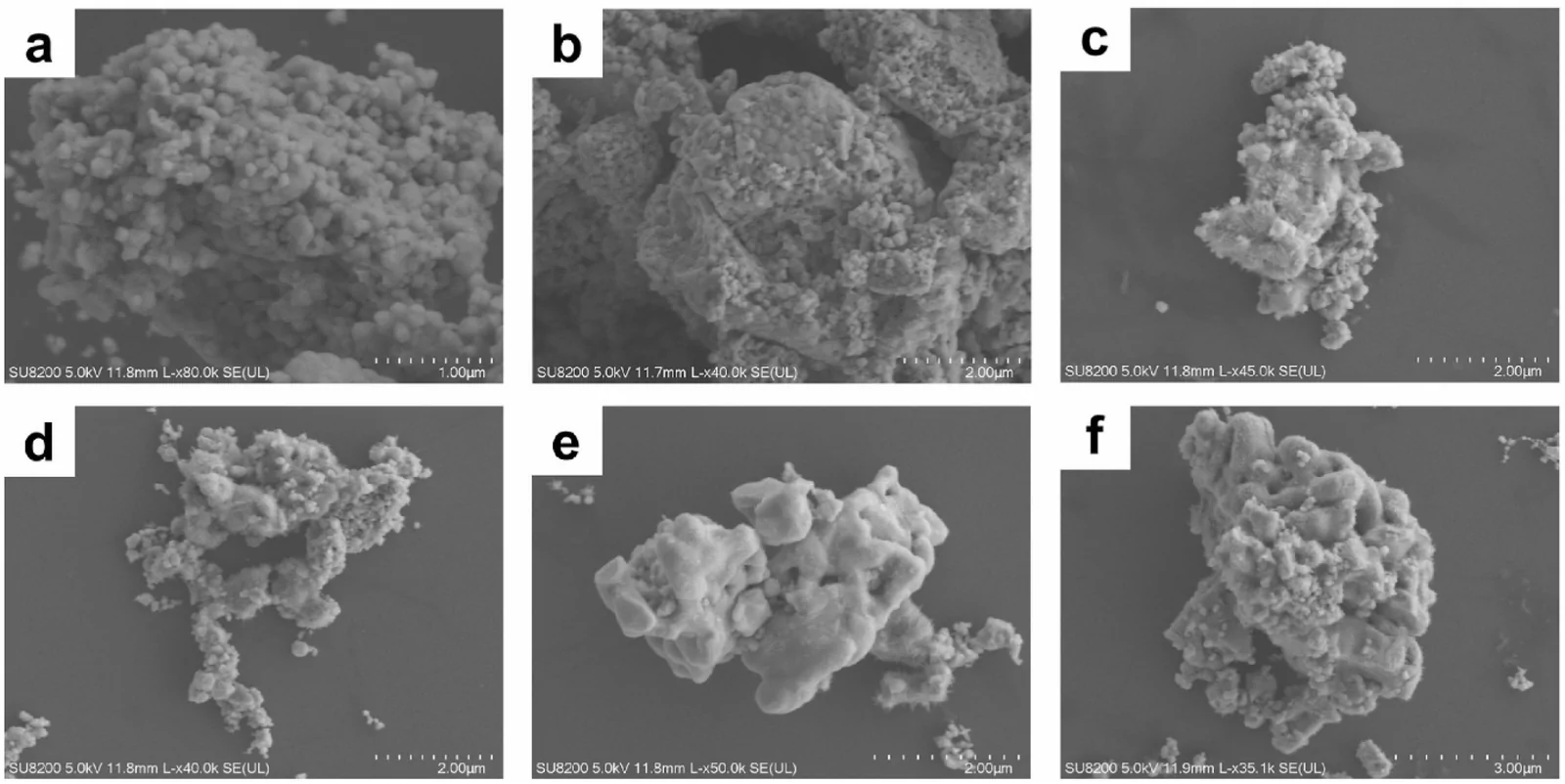
Shows the SEM images of (a) ZrO_2_, (b) ZP_0.1_, (c) ZP_0.2_, (d) ZP_0.3_, (e) ZP_0.4_, (f) ZP_0.5_.^99^

Liu *et al.* analysed different characterization techniques that have been applied to study the structural characteristics of heterojunction photocatalysts based on ZrO_2_. In Fig. 5 SEM results show a spherical morphology for ZrO_2_, while the other morphologies seem to indicate embedding of particles in ZrO_2_. At low doping concentration (ZP_0.1_), only the ZrO_2_ morphology is visible because Pr_6_O_11_ is fully encapsulated by ZrO_2_. As the Pr^3+^ doping ratio increases further, the two phases become embedded within one another, forming a heterojunction structure. The UV-Vis diffuse reflectance spectroscopy shows that pure ZrO_2_ absorbs ultraviolet light below 327 nm. However, by using Pr_6_O_11_, the absorption extends further into the visible region. The bandgap analysis reveals the doped material, indicating the efficient use of visible light due to a narrower bandgap and improved photocatalytic activity. From the band alignment, it is evident that the bandgap of ZrO_2_ is composed of both the valence band and conduction band of Pr_6_O_11_. Since the structure of Pr_6_O_11_ is staggered, the bandgap of the composite is effectively tuned through the material. This helps separate photogenerated electron–hole pairs and facilitate charge transfer.^99^

Jawhari investigates the synthesis of the ZrO_2_–CdWO_4_ nanocomposite. The UV-visible spectroscopy verified the formation of nanoparticles and assessed the bandgap energy. Pure ZrO_2_ and CdWO_4_ had absorption edges at 228 nm and 255 nm, while the nanocomposites showed an absorption edge at roughly 240 nm, indicating a blue shift associated with a smaller particle size and a narrowed bandgap of 2.66 eV. The valence band and conduction band edge positions were calculated based on the estimated bandgap and electronegativity values. The formation of the Type I heterojunction, which aids in charge separation and prevents electron–hole recombination, has been confirmed through these energy levels. XRD revealed crystallinity and phases, revealing zirconia in a tetragonal structure and cadmium tungstate in a monoclinic structure. Crystallite sizes were estimated to be about 26 nm for CdWO_4_ and 8 nm for ZrO_2_. The separation of charges and recombination kinetics of photogenerated charge carriers at the heterojunction interface were determined through PL spectra analysis. Compared to the pure ZrO_2_ and CdWO_4_ semiconductor samples, the ZrO_2_–CdWO_4_ heterojunction exhibited very low PL intensity. Since the ZrO_2_ was coated on the surface of CdWO_4_, there is a less recombination rate of the electron–hole pair due to the low PL intensity. This is because the heterojunction photocatalyst is better than the pure oxides in photolysis activity.^100^

Wahba *et al.* observed that the characteristics of the ZnO and ZrO_2_, as the concentration of ZrO_2_ increased, the intensities of the tetragonal peaks of ZrO_2_ rises correspondingly, showing that the formation of the heterostructure occurred without any secondary phases and impurities. These heterostructures have an average crystalline size within 45–51 nm, suggesting the development of tiny, nanoscale composites that are advantageous for photocatalytic applications. The nanocomposites are mostly composed of nanospherical particles, with a rod-like features, according to the SEM results. As the percentage of ZrO_2_, increases, the morphology, which includes particle size and homogeneity, becomes better.^101^

Basaleh *et al.* study identified that pure ZrO_2_ has a monoclinic phase, matching typical (*hkl*) planes. HR-TEM showed clear crystalline interfaces with well-defined interplanar spacing of monoclinic ZrO_2_ (0.30 nm) and cubic CdO (0.27 nm). By BET surface analysis, it was found that the mesoporous texture still existed even though the loading of CdO nanoparticles resulted in a minor decrease in the surface area due to pore blocking. This ensures that there is a sufficient number of active sites on the catalyst surface for adsorption purposes. The analysis of the atrazine degradation and formation of the products of mineralization, such as nitrate ions (NO_3_^−^) and chloride ions (Cl^−^), was carried out using liquid chromatography methods. Moreover, post-reaction analysis of the used catalysts was performed by means of X-ray diffraction (XRD) and X-ray photoelectron spectroscopy (XPS). It was found that there were no changes in the catalysts' structure and chemical composition. For materials to be recyclable and used in industry, chemical and photophysical stability are essential. ZrO_2_ is renowned for its exceptional stability and biocompatibility. The inherent stability of ZrO_2_, along with the use of CdO, leads to an increase in photocatalytic efficiency. Despite being referred to as facile, the synthesis process entails a number of steps, which complicates industrial application of the catalysts.^102^

Yu *et al.* conducted a comprehensive characterisation of AgCl@Zr^3+^–ZrO_2_ nanostructures using a range of techniques to examine their properties. The confirmation of Zr^3+^–ZrO_2_ phase formation and the addition of AgCl were determined by XRD to establish the heterojunction that drives efficient charge separation. The BET surface areas for Zr^3+^–ZrO_2_ clusters and AgCl@Zr^3+^–ZrO_2_–11 heterojunctions were determined to be 172.75 and 62.03 m^2^ g^−1^, respectively. This high surface area increases the surface photocatalytic reactions due to the availability of numerous reactive sites and strong adsorptive ability. The heterojunction exhibits low PL emission and the smallest EIS arc radius, revealing considerably higher charge separation and lower charge migration resistance at interfaces. Mott–Schottky plots contributed to establishing the band structures and verifying the *S*-scheme charge transfer pathway.^103^

Das *et al.* found that the heterojunction systems based on ZrO_2_, especially the system with ZrO_2_@chitoson composite, are analysed through several methods. The peak broadening in the composite has been observed through the use of X-ray diffraction, which shows that uniformly small ZrO_2_ particles have been formed in the chitosan. Effective bonding of ZrO_2_ and CTS has been confirmed through high-resolution transmission electron microscopy analysis showing well-defined lattice fringes corresponding to (111) planes of monoclinic ZrO_2_. From zeta potential test, it is evident that the composite has a positive charge which helps facilitate the adsorption of negative charged contaminants for their photocatalytic decomposition. The optical bandgap of ZrO_2_ composite is much lower (1.8 eV) than ZrO_2_ (3.2 eV) and CTS (2.5 eV), making it more absorptive of visible light. The reduction in the recombination rate of the photogenerated charge carriers in the composite material is established through the use of photoluminescence (PL) and electrochemical impedance spectroscopy (EIS) studies, implying enhanced efficiency of photocatalytic reactions due to the superior separation of photo-excited charge carriers.^104^

## 8. Photocatalytic degradation performance

**Fig. 6:**
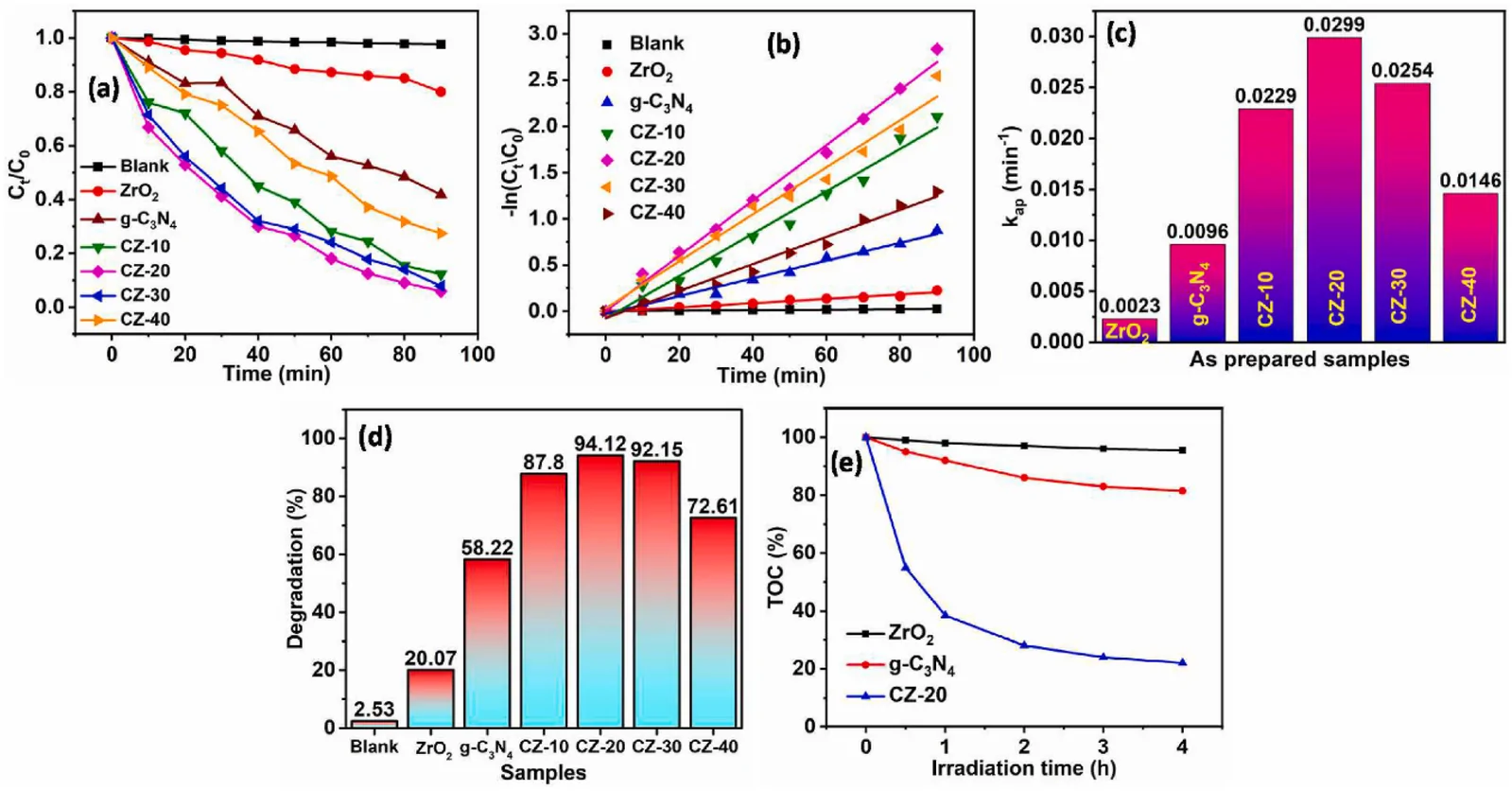
Shows photocatalytic degradation of RhB under natural sunlight. (a) *C*_*t*_/*C*_0_*vs.* time curve, (b) first-order kinetic curve, (c) rate constant of samples for RhB degradation, (d) degradation efficiency of all samples, and (e) TOC elimination % of different samples.^105^

Chand *et al.* elucidated that when ZrO_2_-based heterojunctions are exposed to visible light, their photocatalytic activity increases, particularly for degrading organic pollutants such as Rhodamine B. The degradation rate is measured based on the reduction of its concentration under illumination by sunlight. The rate constant of ZrO_2_–g-C_3_N_4_–20 is 0.0299 min^−1^. The efficiency of mineralization is determined using TOC measurement; for instance, after 1.5 hours, the composite degraded 94.12% of RhB while removing only 66% of TOC showed in Fig. 6. Enhanced charge separation in the heterojunctions, reduced recombination of electron–hole pairs, and enhanced visible light absorption due to the favourable band structure of g-C_3_N_4_ and ZrO_2_ are the reasons behind the enhanced photocatalytic activity.^105^

According to Ramamoorthy *et al.*, the ZrO_2_–TiO_2_ nanocomposite is used to degrade the Alizarin Yellow dye by 90–93%, due to the strong bonding interactions between Zr–O–Ti that decreased the number of surface defects, increased the charge separation efficiency, and formed shallow trap states for photogenerated carriers. The photocatalytic reaction occurred *via* a Type I heterojunction, during which the electrons were moved from the conduction band of ZrO_2_ (−1.1 eV) to that of TiO_2_ (−0.19 eV), leading to the formation of O_2_˙^−^ and OH radical species that initiated dye degradation.^106^ On the contrary, Fikiri *et al.* showed that TiO_2_@ZrO_2_ hybrid nanotubes show a higher level of methyl orange dye degradation of 94.6%. This was attributed to the strong interactions between the two materials, the red shift in UV-vis spectra, FTIR intensity changes, and XRD diffraction peak shift. Moreover, TiO_2_@ZrO_2_ hybrid nanotubes possess high electrical conductivity, higher active sites, higher catalyst dose, as well as excellent reusability, retaining 91% of photocatalytic activity after three consecutive cycles, while exhibiting a slight decrease in the apparent rate constant from 28.36 × 10^−3^ min^−1^ to 27.16 × 10^−3^ min^−1^.^107^

Meena *et al.* synthesised MnFe_2_O_4_/ZrO_2_ nanocomposites exhibit excellent activity as a photocatalyst because of their combined effect and interface interactions, which enhance the ability of separating electron–hole pairs and absorb light. Within 90 min under natural sunlight illumination, the composite has decolourized up to 95% of Methylene Blue dye. The optimum pH value is 7 for the best performance since there will be a reduction when the pH is either too high or too low, owing to OH radical scavenging and inability to generate at low pH values. Ideal amounts for the catalyst and MB dye are 60 mg and 20 ppm, respectively.^108^

Mohammad Zarei synthesized the nanocomposite ZrO_2_/g-C_3_N_4_*via* an ultrasonication process. The performance of photocatalysis improved due to a substantial increase in surface area from 51.8 m^2^ g^−1^ in pure g-C_3_N_4_ to 65 m^2^ g^−1^ in 30 wt% of ZrO_2_/g-C_3_N_4_ composite. When compared to pure g-C_3_N_4_ (0.0114 min^−1^) and pure ZrO_2_ (0.0071 min^−1^), the rate constant of photodegradation of 4-CP attained the maximum value of 0.0173 min^−1^ in the composite materials.^109^ Jalil *et al.* reported CuO/ZrO_2_ by a simple and facile method. CuO/ZrO_2_ nanocomposites have shown excellent photocatalytic degradation ability under UV light, where 96.4% of tetracycline was degraded in 120 minutes. Different levels of concentration and pH have been used to control the photocatalytic activity; the maximum efficiency was achieved by using 0.8 g L^−1^ of the catalyst and pH 12. The high pH level would help to create an effective interaction with the negatively charged tetracycline because the nanocomposite is positively charged at higher pH. Also, the performance of CuO/ZrO_2_ is better than that of ZrO_2_ due to absorption of visible light.^110^

Density functional theory (DFT) calculations play a very significant role in understanding the inherent electrical properties of materials in photocatalysis, and it guides the subsequent study process. Through the link between semiconductors and their catalytic processes, DFT plays an important role in solving the problems presented in the experiments. Understanding the interactions between photocatalysts and substrates and the impact of defects is possible through DFT by predicting their electronic structures, surface reactivity, and charge transfers. The design of efficient photocatalysis is made easier by some DFT calculations like band structures, density of states, optical properties, and charge distribution.^111^

AI-based algorithms play a key role in optimizing the photocatalytic decomposition of pollutants such as tetracycline, in particular gradient boosting regression (GBR), ridge regression (RR), and particle swarm optimization (PSO). Such algorithms help to increase the precision of predictions, simplify the mechanisms of reactions and deal with the complexity of experimental data in an effective manner. Although RR deals with the issues of multicollinearity and overfitting and is reliable and relatively easy, GBR is better at grasping variable interactions. PSO helps to find the optimal set of parameters for increasing removal efficiency and minimizing costs. The use of AI makes the understanding of reaction mechanisms simpler and hence leads to more secure and economic systems. Future studies will focus on considering more variables in AI algorithms.^112^

The photocatalytic efficiency of Ce–ZrO_2_–GO nanocomposites for eosin yellow dye decomposition under visible light is demonstrated by Samuel *et al.* study. Owing to the synergistic effect of GO sheets and Ce^3+^ ions, the rate of degradation of the 0.3% Ce-doped composite is 15.98 times higher compared to the pure ZrO_2_. Based on DFT calculations, the bandgap energy of Ce–ZrO_2_–GO (2.81 eV) is lower than that of pure ZrO_2_ (5.01 eV). Although GO sheets increase the lifetime of charge carriers, Ce^3+^ ions enhance mobility and reduce recombination. Ce–ZrO_2_–GO can be used as an efficient photocatalyst for decomposition of organic pollutants due to its stability in repeated cycles and the capability to reach 76% mineralization of EY dye within five hours.^113^

The photocatalysts, particularly those based on ZrO_2_ and consisting of lanthanide-doped ZrO_2_/g-C_3_N_4_ nanocomposites, used for purification from pollution necessitate DFT investigation. This involves a plane-wave basis set, optimized pseudopotentials, as well as certain *k*-point sampling for energy convergence. van der Waals corrections, hybrid density functionals for electrical characteristics, and AIMD calculations to study the thermal and dynamical stability are among other important aspects of DFT methodology. Band structure calculations indicate the presence of a direct band gap of about 5.46 eV and 2.70 eV for ZrO_2_ and g-C_3_N_4_, correspondingly, while structural optimization indicates the favourable energy configuration. Charge transfer analysis reveals considerable charge rearrangement at the interface between the two materials, thus confirming an efficient electron–hole separation, an essential parameter for photocatalysis. In general, DFT aids in designing efficient photocatalytic processes for environmental purposes.^114^

Degradation efficiency of present-day zirconium oxide-based nanocomposite systems is quite high, reaching on average 89.56% and often surpassing 75% under optimal conditions. The efficiency of charge separation using advanced strategies like heterojunction engineering is linked to better performance. Higher degradation efficiency is shown by heterojunction zirconium oxide-based nanocomposites, with doxycycline degradation efficiency reaching 98.8%. Photocatalytic degradation is carried out with the help of crucial reactive oxygen species such as hydroxyl radicals, which require optimal conditions, especially around neutral pH. Currently, there are issues in commercialization of zirconium-based nanocomposite systems despite promising laboratory findings, including low technological readiness (TRL 1–4), instability and reusability problems, and complexity in treating real pharmaceutical effluents. Comparing the studies directly is difficult due to standardization challenges and the necessity for comprehensive techno-economic assessment. AI and advanced modelling can be incorporated in future research to increase development efficiency.^115^

## 9. Conclusion and future research directions

The current review emphasizes the importance of photocatalysis, which is a promising and environmentally friendly technique in tackling water pollution. There are many semiconductor catalysts; however, ZrO_2_ has gained a lot of attention owing to its high chemical stability, non-toxicity, and excellent oxidation ability. The wide bandgap of ZrO_2_ has been identified as one of its drawbacks when it comes to light absorption in the visible region. Recently, there have been efforts to design heterojunctions of ZrO_2_ to address the above issue. However, despite the substantial progress achieved in ZrO_2_-based photocatalysts and the development of their composites for environmental purposes, several obstacles currently impeding their application need to be addressed. The major one lies in the lack of large-scale applicability, since the studies of these catalysts and their reusability under continuous operation have not been sufficiently investigated. The problem of non-standardized test conditions is also of considerable importance. Different catalyst amounts, pollutant concentrations, light intensity, wavelength, and reaction conditions do not allow making comparisons between various studies. Moreover, most photocatalytic experiments are performed on synthetic dye solutions, while studies concerning wastewater containing various pollutants are quite rare.

The progress that has been made so far in the field of photocatalysis by using ZrO_2_ as a semiconductor and how the design of the heterojunction structure can help in overcoming the shortcomings associated with the use of this material as a photocatalyst under visible light are outlined in this paper. While the chemical stability of this material in adverse conditions and lack of toxicity to the environment make ZrO_2_ a suitable material for use as a photocatalyst, its inefficiency under visible light irradiation can be attributed to its wide bandgap and high electron–hole recombination rate. The synthesis of a wide variety of materials used to create ZrO_2_ heterostructures has been observed to be composed of narrow bandgap semiconductors and carbon-based compounds. As a result, more visible light absorption has been achieved, leading to greater charge separation and charge transport. The shift from type-II heterojunctions to the *Z*-scheme and *S*-scheme heterostructures is also crucial in achieving better photocatalytic properties.

Improved light utilization, charge separation, charge transfer at interfaces, and heterojunction design substantially increase the efficiency of photocatalytic activities of ZrO_2_-based catalysts, enabling their role in environmental remediation. However, even with the extensive progress achieved through research and development, several challenges stand in the way of their wider application. It is essential to conduct investigations into the stability and reusability of ZrO_2_-based heterojunctions under conditions of multiple and prolonged use. Also, there is a lack of information about their ability to perform in real wastewater containing organic substances, competitive ions, and other pollutants, as most of the studies have been carried out on synthetic polluted solutions.

Catalyst preparation on a large scale has difficulties in ensuring material uniformity in large-scale production. Recovery of catalysts from nanopowders can be very difficult, causing possible secondary contamination. Stability and recyclability of the photocatalysts are important since the degradation of their performance will result in a shorter operational life. The complexity of real wastewater, such as the presence of different pollutants and typical interferences, makes results obtained in the laboratory less reliable. Light penetration into large reactors is affected by several parameters, such as turbidity. It is not enough to rely on photocatalytic performance alone when assessing commercialization feasibility. Thus, the direction of future research should be in standard test procedures and long-term stability experiments. The commercial development of ZrO_2_-based heterojunction photocatalysts as a promising material into commercially viable technology for wastewater treatment will be a challenge to overcome in the future. From the results of this review, these novel systems have shown great success in their ability to effectively remove different kinds of pollutants, including organic dye pollutants, toxic metal ion pollutants, and others that are new and emerging pollutants. However, there is also another set of problems related to these systems. The use of this system in natural light conditions, for instance, is one of the challenging areas that require further investigation.

In future work, the optimization of the enhanced photocatalytic heterostructures should be conducted. Processes such as defect engineering and heterojunction formation will be one of the methods to enhance the efficiency of photocatalytic degradation. Regarding future concerns, real wastewater was also tested using the catalyst. It can thus be concluded that the development of ZrO_2_-based heterojunction photocatalysts activated by visible light is a very promising area in which research can be done to bring about breakthroughs in future water treatment technology. It may have a significant contribution towards the design of an environmentally friendly system for water purification process.

## Author contributions

Sivasundhari Sivakumar: writing – original draft, visualisation, validation, and conceptualization. E. James Jebaseelan Samuel: writing – original draft, review and editing, validation, supervision, conceptualization.

## Conflicts of interest

The authors state that no competing interests exist regarding the publication of this manuscript.

## Data Availability

No new data were created or analyzed in this study. Data sharing is not applicable to this article.
